# *In silico* docking studies for designing potent anti-diabetic derivatives of swertiamarin with enzyme HMG COA reductase

**DOI:** 10.1186/1755-8166-7-S1-P97

**Published:** 2014-01-21

**Authors:** Jayshil Bhatt, Hitesh Vaidya, Varun Khanna, Naisargee Patel, Ramesh Goyal

**Affiliations:** 1 Memorial University of Newfoundland, St. John’s, Canada; 2 Institute of Life Sciences, Ahmedabad University, Ahmedabad, India

## Background

Swertiamarin, a secoiridoid glycoside is found in abundant quantity in *Enicostemma Littorale* herb and is the main constituent responsible for anti-diabetic and anti-obesity effects of the plant extract. It has been reported to act on various enzymes and transcription factors involved in glucose and lipid metabolism, including inhibition of the enzyme HMG-CoA reductase which might be one of the mechanisms responsible for the antihyperlipidaemic activity. However, owing to its high water solubility, it has a low plasma half life; and thus we have designed its derivatives which bind more efficiently with HMG CoA Reductase.

## Materials and methods

Docking was carried out using software namely Autodock and Vina, and repeated at least thrice to minimise the error and to confirm the repeatability. The obtained results were scored and sorted on the basis of the binding energy. The Autodock docking was only done on the inhibitor ligands that were taken from the crystal structure to cross check the results of Molecular Operated Environment [MOE] in terms of binding orientation. Attempts were also made to evaluate *in silico* toxicity and identification of other possible targets for the lead molecule swertiamarin.

## Results

We could design 23 compounds which were docked into the active site of the crystal structure of HMGR. The interactions of these molecules were compared with the presently known inhibitors such as atorvastatin and simvastatin. Based on the results of docking score a number of potent ligands for the enzyme HMGR as compared to swertiamarin were established. It was observed that the designed molecules SWL18, SWL21, SWL22 showed better docking score (-60.74; -61.43 and –55.68 respectively) and tight binding in pockets. Docking score of these molecules were very similar to the atorvastatin and simvastatin.

## Conclusions

SWL18, SWL21, SWL22 possess tight binding with HMGR as compared to swertiamarin, which suggest that these molecules could have better HMGR inhibition.

